# A Strong Seasonality Pattern for Covid-19 Incidence Rates Modulated by UV Radiation Levels

**DOI:** 10.3390/v13040574

**Published:** 2021-03-29

**Authors:** Christos Karapiperis, Panos Kouklis, Stelios Papastratos, Anastasia Chasapi, Antoine Danchin, Lefteris Angelis, Christos A. Ouzounis

**Affiliations:** 1 School of Informatics, Aristotle University of Thessaloniki, GR-54124 Thessalonica, Greece; c.karapiperis@gmail.com (C.K.); lef@csd.auth.gr (L.A.); 2 Chemical Process & Energy Resources Institute, Centre for Research & Technology Hellas (CERTH), Thermi, GR-57001 Thessalonica, Greece; stelios.papastratos@aveva.com (S.P.); chasapi@certh.gr (A.C.); 3 Laboratory of Biology, Medical School, University of Ioannina, GR-45110 Ioannina, Greece; pkouklis@uoi.gr; 4 Department of Biomedical Research, Institute of Molecular Biology & Biotechnology, Foundation for Research & Technology Hellas (FORTH), GR-45115 Ioannina, Greece; 5 Kodikos Labs, F-69007 Lyon, France; antoine.danchin@normalesup.org; 6 Institut Cochin, F-75013 Paris, France

**Keywords:** Covid-19, seasonality, ultraviolet radiation, machine learning

## Abstract

The Covid-19 pandemic has required nonpharmaceutical interventions, primarily physical distancing, personal hygiene and face mask use, to limit community transmission, irrespective of seasons. In fact, the seasonality attributes of this pandemic remain one of its biggest unknowns. Early studies based on past experience from respiratory diseases focused on temperature or humidity, with disappointing results. Our hypothesis that ultraviolet (UV) radiation levels might be a factor and a more appropriate parameter has emerged as an alternative to assess seasonality and exploit it for public health policies. Using geographical, socioeconomic and epidemiological criteria, we selected twelve North-equatorial-South countries with similar characteristics. We then obtained UV levels, mobility and Covid-19 daily incidence rates for nearly the entire 2020. Using machine learning, we demonstrated that UV radiation strongly associated with incidence rates, more so than mobility did, indicating that UV is a key seasonality indicator for Covid-19, irrespective of the initial conditions of the epidemic. Our findings can inform the implementation of public health emergency measures, partly based on seasons in the Northern and Southern Hemispheres, as the pandemic unfolds into 2021.

## 1. Introduction

*Problem*: One of the most crucial questions for the management of the Covid-19 pandemic has been whether the epidemic would exhibit a consistent seasonality pattern [1]. As the first cases started spreading globally in early 2020, there was limited seasonal data through which this pattern could in fact be detected [2]. Yet, from the very early days, the question has lingered in the literature and remained partly unanswered [3,4]. Many studies have focused on temperature or humidity effects that might slow down viral transmission, with rather poor results (for references, see previous [2]). The community’s attention to those parameters can partly be explained by our own perception of seasons, essentially following anthropocentric expressions such as “the winter blues” or “a summer feeling”. The plurality of those studies and their significant coverage by the media, given that the effects appeared marginal, have discredited the argument for a seasonality element of Covid-19 [5] and mistakenly led to appeals for caution to deal with these “misconceptions” [6].

*Background*: As the pandemic has engulfed the globe, affecting human activities on an unprecedented level, and concerns that Covid-19 may become endemic in the entire population, as other coronavirus infections have [4], the issue of seasonality must be unambiguously settled, especially as we are now approaching a full year of the epidemic expansion [7]. Other factors such as population density [8] or public health measures [9] have been seen as critical and thus challenged notions of seasonality of Covid-19, intertwined with irregular seasonal parameters such as humidity and air temperature [10]. Yet, recent research points to strong seasonal forcing of respiratory diseases that are modulated by environmental factors [11], an element that supports a similar cycle for Covid-19 [12]. Furthermore, after decades of seasonality research on the influenza epidemic [4], there are indications for regularities affected by seasonal fluctuations connected with latitude, at least in certain regions [13]. Finally, we know very little about coronavirus infections of other species in the wild and the behavioral or the environmental factors that reduce virus spread, potentially connected with solar exposure, for instance in the case of sea bird colonies in the southern Indian Ocean [2].

*Ultraviolet*: The realization that ultraviolet (UV) radiation modulates the infection rate and therefore the spread of the epidemic has grown only recently, based on previous lab studies [14]. This has been achieved by either using preliminary evidence from high-altitude locations as proxies for latitude values and thus UV levels before a complete yearly cycle [2] or sophisticated numerical models with predictive capabilities that take UV levels into consideration [7]. Detailed forward simulations based on the first half of 2020 indicate that UV levels may influence Covid-19 growth rates [15]. These studies have provided the first compelling evidence for seasonality of Covid-19 connected to UV levels as the ultimate parameter that signals seasons or day length, concurrently for winter/summer cycles in the Earth’s hemispheres.

*Causality*: Whether UV is just a factor that indicates seasons (far better than temperature or humidity, and generally “weather” patterns) or influences the biology of the virus (transmissibility in the open) or the host (e.g., vitamin D, other immune regulation) is less relevant. There are multiple reports that link vitamin D with UV radiation [16], and the potential effects in a clinical Covid-19 setting [17] that can be one element of the UV seasonality pattern [18]. Correlations with latitude in European countries and vitamin D (25-hydroxyvitamin D) further support the evidence [19] and drive the argument for a deeper and global analysis. Thus, despite our limited knowledge for the underlying virus–host mechanisms, the quest for seasonality parameters at play that can describe or even predict Covid-19 growth is of paramount importance.

*Motivation*: As the world needs nonpharmaceutical interventions (NPIs) to delay virus spread and contain outbreaks at the national, state or local level [20], a careful consideration for seasonality will become a key factor to mitigate the damage created by social distancing and lockdown regimes [7]. Policymakers and the public will need a deeper appreciation for better NPIs that not only are hugely cost-effective but follow the natural cycles of the planetary clock and weigh against short-term, clumsy and longer-term, damaging containment measures for human activity and the world economy, also negatively affecting environmental protection. If a seasonality pattern for Covid-19 is confirmed, it follows that imposing or lifting restrictive measures should take into account relevant factors or, in the absence of those, replace them with artificial substitutes, at least for critical infrastructures such as transport or public spaces.

*Seasonality*: In this work, we examine Covid-19 incidence rates and attempt to correlate them with social behavior data and seasonal cycles of UV radiation levels across twelve countries. The selection of those countries was based on latitude, geography, comparable demographic or economic indices, epidemic surveillance statistics and availability of UV recordings over a yearly cycle. We showed that the UV level dominated the seasonal pattern of the epidemic and modulated the incidence rate for Covid-19, independently of other parameters, thus providing a well-defined factor to assess seasonality patterns of past, present or future epidemic outbreaks [21].

## 2. Materials & Methods

### 2.1. Country Filter Parameters

A list of all world countries was the initial input for target country selection. Steps of data harvesting are listed below. Absolute and relative coastline measures were recorded (length and length/area), reflecting both size and exposure to ocean climate. Socioeconomic indices included literacy levels (percent of population) and density (population/area), as well as GDP in 2019 (per capita). Epidemic surveillance statistics included Covid-19 tests (absolute: tests and relative: tests per million people). We defined all the above features as “filter” parameters, in that they played no further role as inputs in the ensuing correlation and learning models. The structure of the input data is provided as an example, where the country features are recorded as filter parameter vectors and include geo- or socioeconomic indices, as above (Table 1). Deploying UV radiation level statistics as an additional criterion (a “model” parameter, see below) for further pruning of the initial list resulted in a shortened list of 80 countries based on UV data availability and no extra numerical thresholds. To avoid size effects, 29 countries with population sizes of <1 million or >1 billion inhabitants were excluded.

### 2.2. Selection of Target Countries

Subsequently, 51 country filter parameter vectors were clustered using k-means (in R), to identify countries with similar statistics, and at least some pairwise similarity to one other country (excluding total outliers). We omitted countries with a large early spread of the epidemic, such as Italy or Spain. The final selection aimed at the detection of 12 target countries, based on the above criteria. The epidemiological statistics (incidents, daily cases reported) and their potential relationship to UV radiation or mobility (walking, driving), which we defined as “model” parameters, did not play a role in country selection, other than data availability (for UV radiation rates). The only other element in the choice of target countries was the location across hemispheres, with six countries in the Northern Hemisphere, two countries near the equator and four countries in the Southern Hemisphere, a total of 12 target countries in three distinct, nonoverlapping groups (Table 2), similar in spirit to a North-equatorial-South comparative study [5]. For the North–South, we “paired” the most similar countries as follows: Canada–Australia, Japan–South Africa (large countries, Group 1) and Austria–New Zealand, Norway–Chile, plus two more countries (Greece, Ireland) to enrich the “north” data group (smaller countries, Group 3). The quasi-equatorial countries were the control (Group 2).

### 2.3. Country Model Parameters

Epidemiological statistics were imported directly as reported, during the period 1 January 2020 to 30 November 2020 [22]. While in most cases from the selected 12 countries the data are regularly reported, there was a single exception for Chile on 16 June 2020. Then, the Ministry of Health announced 31,422 backdated cases included in that specific daily report. We have not smoothed the daily counts and made a choice to keep raw data, despite known, or indeed other unknown, incidents. This choice was guided by various other human factors, such as super-spreader events or other accidents, or cruise ship outbreaks early during the epidemic [23].

Mobility statistics were obtained by Apple Covid-19 mobility trends during the entire period examined (starting 21 January 2020). Apple Covid-19 mobility trends are reports published daily and reflect requests for directions in Apple Maps for driving and walking activities [8]. A technical issue for 23/24 May was resolved by using a three-day rolling average of previous dates to fill these records. These data are evidently crucial for correlation with incidence rates and directly reflect the imposition of restrictive measures which vary from country to country, irrespective of the level of compliance across the population. Population coverage was by definition incomplete: these data were used as a per-country global indicator of the efficiency of restrictive measures.

Ultraviolet (UV) radiation levels were downloaded from the Tropospheric Emission Monitoring Internet Service (TEMIS) archive [24]. We directly used DNA damage UV dose (UVDDF) and vitamin D index UV dose (UVDVF), both measured in kJ/m^2^. We opted to focus on those and not on the much simpler UV index that has a more limited numerical range.

### 2.4. Feature Scaling and Comparisons

Model parameters were either correlated to each other as raw values in logarithmic scale or as dependent, normalized variables with the independent time variable, reported in days. In the latter case, feature scaling with min-max normalization was implemented for comparability and avoidance of incompatibility for extreme (very small or large) values, as follows: x′ = (x − min(x))/(max(x) − min(x)); where x is m for mobility, p for cases or r for radiation. Empirical comparison of the distributions for p (cases) and r (radiation levels) was performed by computing the absolute difference Δ i = |p′i − r′i| for each country i. Finally, to obtain a single metric of descriptive statistics for each country case, sums of squares (SSQ) Δ i2 of the difference distributions were computed.

### 2.5. Heat Map Visualization

We applied PCA analysis and analyzed data for the 12 countries on cases per 1 million population, mobility data related to driving and walking with UVDDF to find the correlation between incidence rates and UV radiation levels. Dimensionality reduction by PCA transformed the data by projecting to a set of orthogonal axes and visualized by heat map representations. We used ClustViz [25] with model parameters for each country normalized within observed values without further clustering. These parameters for each country were, as stated, daily cases, mobility data, UVDDF and UVDVF, across the independent time variable shown in days.

### 2.6. Machine Learning Models and Feature Importance

We utilized automated machine learning (ML) for time series data to find the best model for predictions and extract evidence for feature importance. The ML optimization process, with a multitude of models that need to be tuned for optimal performance and a complex choice of hyperparameters, model types and configuration details, was executed on Microsoft Azure Automated ML for all target countries. Thus, the resulting optimization solution space could be reduced in complexity by the choice of few models among many potential solutions. Experiments for Greece, for instance, resulted in 1921 runs, and the best model was deemed one “voting ensemble”. The cloud service AzureML (“interpret”) was used to implement model interpretability techniques developed by Interpret-Community (see https://github.com/interpretml/interpret-community/, accessed on 18 February 2020), an open-source Python package for training interpretable models and explain blackbox AI systems. Meta-explainers automatically select a suitable direct explainer and generate the best explanation based on the given model and data sets. In this case, we use the tabular mimic explainer lightGBM. A mimic explainer is based on training global surrogate models (https://christophm.github.io/interpretable-ml-book/global.html, accessed on 18 February 2020) to mimic blackbox models. A global surrogate model is an intrinsically interpretable model that trained to approximate the predictions of any blackbox model as accurately as possible. We interpreted the surrogate model to draw conclusions about the blackbox model, and in this case, to derive support for the hypothesis of UV levels as a seasonality index that modulates Covid-19 daily case dynamics over the reported period in 2020.

### 2.7. Data Sources

Epidemiological: Max Roser, Hannah Ritchie, Esteban Ortiz-Ospina, Joe Hasell: Coronavirus Pandemic (COVID-19). OurWorldInData.org 2020: https://ourworldindata.org/coronavirus, accessed on 18 February 2020.

Mobility: https://www.apple.com/covid19/mobility, accessed on 18 February 2020.

UV radiation: http://www.temis.nl/uvradiation/UVindex.html, accessed on 18 February 2020.

### 2.8. Auxiliary Sources

Ministry of Health, Labour and Welfare. Available from: https://www.mhlw.go.jp/english/, accessed on 18 February 2020.

New Zealand’s elimination strategy for the COVID-19 pandemic and what is required to make it work. Available from: https://www.nzma.org.nz/journal-articles/new-zealands-elimination-strategy-for-the-covid-19-pandemic-and-what-is-required-to-make-it-work, accessed on 18 February 2020.

“Team of 5 million” has achieved the lowest COVID-19 death rate in the OECD—but there are still gaps in our pandemic response. Available from: https://blogs.otago.ac.nz/pubhealthexpert/2020/07/22/nzs-team-of-5-million-has-achieved-the-lowest-covid-19-death-rate-in-the-oecd-but-there-are-still-gaps-in-our-pandemic-response/, accessed on 18 February 2020.

Unemployment rate. Available from: https://www.stats.govt.nz/indicators/unemployment-rate, accessed on 18 February 2020.

### 2.9. Patient and Public Involvement

No patients were involved in this study.

## 3. Results

We investigated the reported Covid-19 incidence rates for twelve carefully selected countries with similar characteristics in terms of geography and socioeconomic factors, appropriately distributed around the globe (see Methods, Table 1). The most similar countries according to their features (“filter parameters”) but at opposite hemispheres could be considered as pairs that were directly comparable (four pairs, eight in total), enriched by two additional countries in the Northern Hemisphere (as internal controls), and two equatorial countries as controls, in three groups (Table 2). Incidence rates are influenced by human activity, a fact reflected in mobility data released to fight Covid-19 spread and thus taken into consideration in all subsequent analyses to mirror restrictive measures.

To assess seasonality [11], and based on our previous, preliminary results indicating a role for ultraviolet (UV) radiation levels [2], we have coupled mobility data and incidence rates (an obvious choice) with UV data for 2020, until November 30th. Given that respiratory infectious diseases, such as influenza, have been recently shown to be affected by UV levels in certain regions [13] and the lack of support for spurious environmental factors such as humidity and temperature [15,26], we opted to disregard the latter in order to focus on a predictable, oscillating and latitude-dependent symmetrical index to assess seasonality. This critical choice is supported by predictive models, where UV levels are found to strongly correlate with local incidence rates, compared to temperature or humidity, even in a limited time period—until April 2020 [7].

To depict the interplay between physical indices that define seasonality (UVDDF, UVDVF), social behavior reflected in restrictive measures (driving, walking) and the reported Covid-19 incidence rates (collectively defined as “model parameters”), per-country snapshots were derived, in quotidian-independent or -dependent fashion. While regular variation would suggest a seasonality pattern for Covid-19, even exceptions to the rule can be illuminating, explained by historical records, and thus would not challenge the global picture.

**Figure 1:**
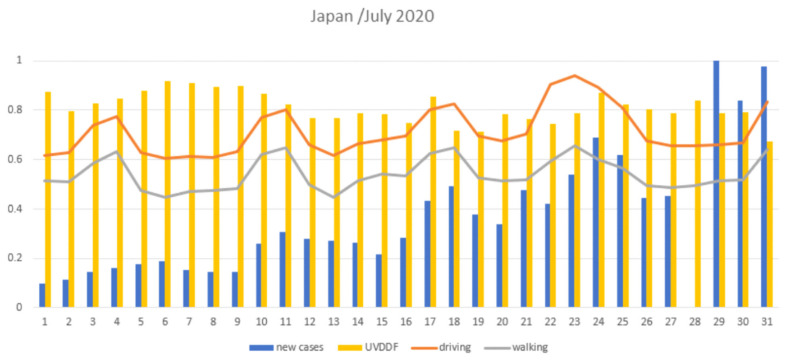
Example of Japan: new cases, driving, walking and DNA damage UV dose (UVDDF) data for July 2020. Day of the month (*x*-axis) and (min-max) normalized values for daily cases (‘new cases’), UV (UVDDF) and mobility (driving, walking), as in legend. Despite an overall high UV level, the surge of Covid-19 cases towards the end of July is evident, as an exception to the rule (see text for details).

For example, in the case of Japan, if Covid-19 was seasonal, one would expect a relatively uniform distribution of incidence rates during July 2020: yet, cases started rising after mid-July. This acceleration resulted in more than 1000 daily cases reported nationwide, in 29–31 July (Figure 1). Specifically, Tokyo recorded 12,691 total cases as of 31 July, of which 6466, or more than half, were reported in July. In the second half of the month, there were numerous days with over 200 cases, and a few with over 300. At the same time, the UVDDF index remained high for July, and mobility values actually increased in that period. The Tokyo Metropolitan Government uses the ratio of positive PCR tests as one of its monitoring benchmarks, which also rose in this period. From around mid-May to mid-June, it remained in the 1–2% range, then increased and, as of July 29, reached 6.6% (see also Methods, auxiliary sources). This surprising surge was probably due to an internal tourism campaign launched a few days earlier, amidst heavy criticism (see https://www.reuters.com/article/us-health-coronavirus-japan-idUSKCN24N0JE, accessed on 18 February 2020). Other factors might have also been at play.

Another rather unique exception is represented by New Zealand: in this case, the country experienced significant outbreaks towards the end of the summer (early March 2020), and quickly shifted to mitigation measures, flattening the observed spread of the epidemic. At the same time, a combination of border controls, favorable geography and a stringent lockdown policy reduced community transmission during the winter period. As a result, New Zealand recorded one of the lowest COVID-19 mortality rates and limited economic damage compared to other high-income countries, so far. Similar success has been recorded in other countries with stringent lockdown policies, especially in Asia.

**Figure 2:**
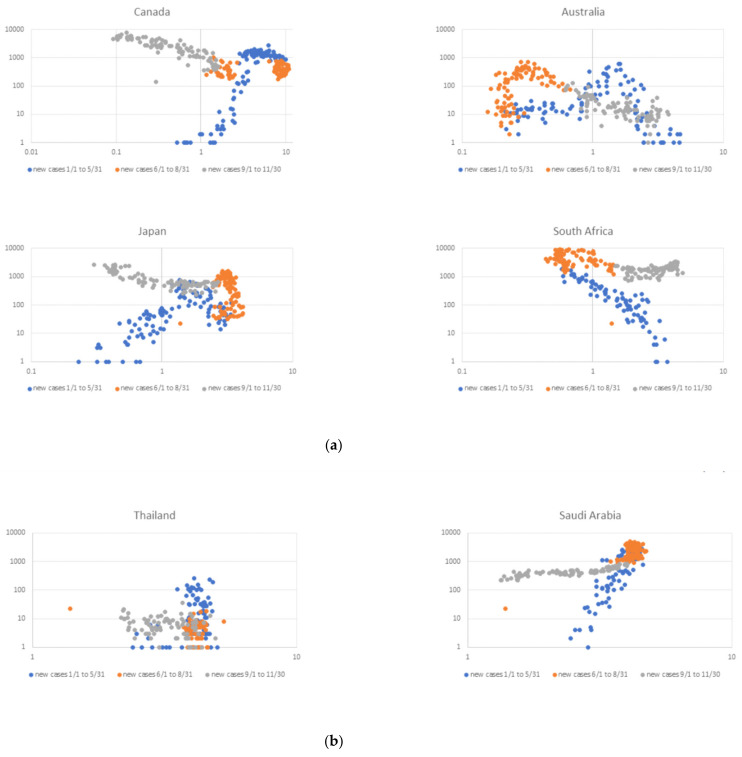

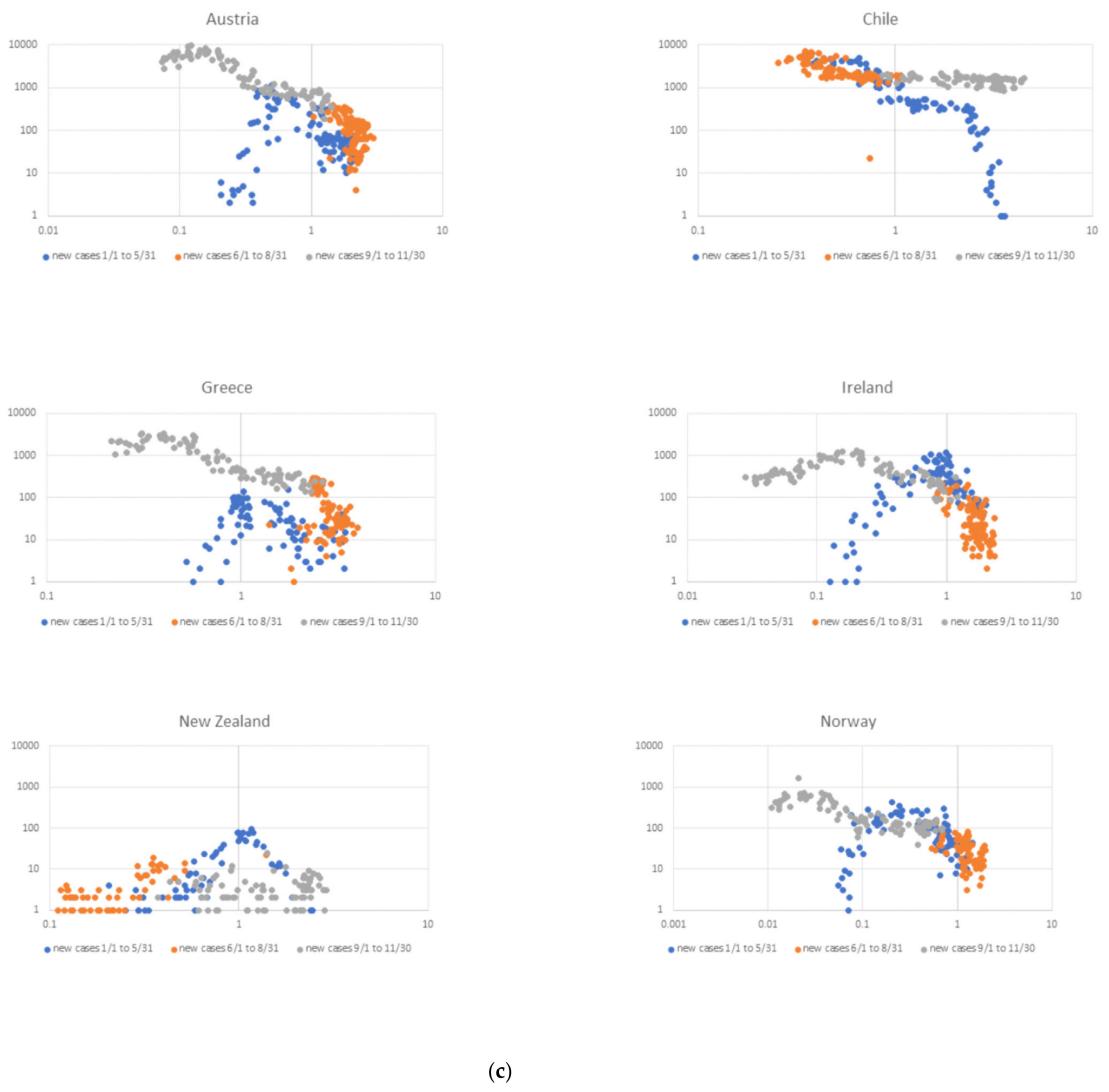
(**a**) Quotidian-independent reporting of case reports vs. UV levels for Group 1 countries. This coarse-grained view shows daily Covid-19 cases (y-axis) and UVDDF levels (*x*-axis), marked by a point, on a logarithmic scale. There are three periods shown as follows: 1 January to 31 May (blue), 1 June to 31 August (orange) and 1 September to 30 November (gray). The range of UVDDF can be obtained by projection onto the x-axis, and differs from country to country. (**b**) Quotidian-independent reporting of case reports vs. UV levels for Group 2 countries, as in Figure 2a. (**c**) Quotidian-independent reporting of case reports vs. UV levels for Group 3 countries, as in Figure 2a.

The above examples, however, say little about seasonality trends throughout almost the entire year: we first examined these trends in a coarse-grained manner by selecting three periods in 2020 and associating UVDDF values with raw Covid-19 rates for the three groups of countries, where North vs. South could be equivalenced by a rotation (horizontal flip) of the UV dimension (x-axis) (Figure 2).

In the first group, Australia—another exceptional case, due to the reported early cruise ship outbreaks—exhibited a high incidence rate towards the end of the summer, i.e., until May 2020 (Figure 2a). In contrast, Canada, in the same “pair”, showed a high incidence rate until May, when it dropped during the local summer and increased in the third period (Figure 2a). Similarly, South Africa started off with low incidence rates, peaked during the second period/local winter and dropped again later (Figure 2a). Again, Japan, its paired counter-example country, and similarly to Canada, started with fewer cases, presented the above-mentioned exceptions in summer and accelerated during the Fall 2020.

In the second (control) group, in the two quasi-equatorial countries with similar filter parameters (see Methods), the seasonality pattern should be dampened during the yearly cycle: again, these patterns were somewhat convoluted and, not surprisingly, imperfect. Again, local incidents or social behavior explained exceptions and reinforced the model (Figure 2b). In the case of Thailand, there was an emergency lockdown that was imposed on 25 March until 30 April, as a reaction to a surge of cases (see https://uk.reuters.com/article/instant-article/idUKKBN21C0GY, accessed on 18 February 2020), due to regional outbreaks in that period. For Saudi Arabia, which experiences a wider variation of UV levels due to its higher latitude, some seasonality was observed, yet daily cases over the summer soared, most likely as a result of indoor, air-conditioned lifestyle in urban locations. As a matter of fact, both trends were independently confirmed by machine learning-based feature extraction (see below).

In the third group, all countries followed a highly regular pattern, except New Zealand, for reasons mentioned above (Figure 2c). The regularity of UVDDF levels contrasted to Covid-19 incidence rates over the three 2020 periods strongly suggested a seasonality pattern modulated by UV, on a coarse-grained level. As pairs, Austria and New Zealand did not appear as regular as expected, however, Norway and Chile displayed a more consistent “mirror-image” pattern, notwithstanding a slight irregularity in the case of Chile in mid-June (see Methods). Remarkably, the two additional countries, Greece and Ireland, showed similar trends to those of Austria and Norway, despite different local conditions, cultures and timing of any restrictive measures (Figure 2c). This original quotidian-independent presentation of environmental vs. epidemiological parameters can be used to broadly assess seasonality and should be applicable to other infectious disease analysis frameworks.

**Figure 3:**
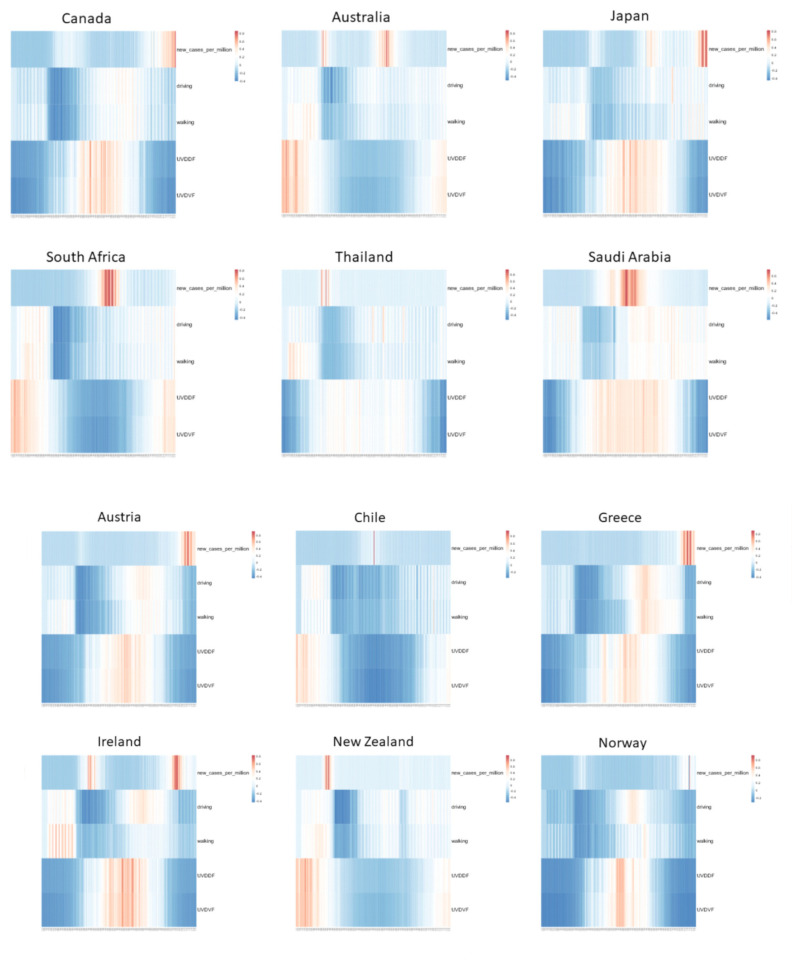
Display of normalized values of model parameters for target countries. The more familiar quotidian-dependent monitoring of model parameters is shown with day of the year (*x*-axis, 1 January to 30 November) and new daily cases, driving, walking, UVDDF and vitamin D index UV dose (UVDVF) are shown as panels (*y*-axis). All values are normalized, color-coded (scale shown, upper right for each case/country), ranging from blue color for negative association to red color for positive association (see Methods).

The quotidian-independent observations, providing a coarse-grained view of the course of the epidemic on a per-country basis (Figure 2), needed to be coupled and assessed against the different restrictive measures that were imposed but not necessarily cross-coordinated for target countries. These measures are reflected by mobility data, available for driving and walking patterns (Figure 3). In particular, for the Northern Hemisphere, initial lockdown regimes were relaxed during May and over the summer, with some opportunity for local or even international travel, patterns that were easily and consistently detectable in the mobility data as walking or driving (requests for map directions). Conversely, for the Southern Hemisphere, the restrictive measures over local winter, i.e., the same period, were stricter, due to rising Covid-19 outbreaks. A heatmap association of model parameters demonstrated relations in a succinct manner. At the same time, UVDDF and UVDVF values were highly correlated, as expected, and followed the Covid-19 daily case reports (Figure 3).

**Figure 4:**
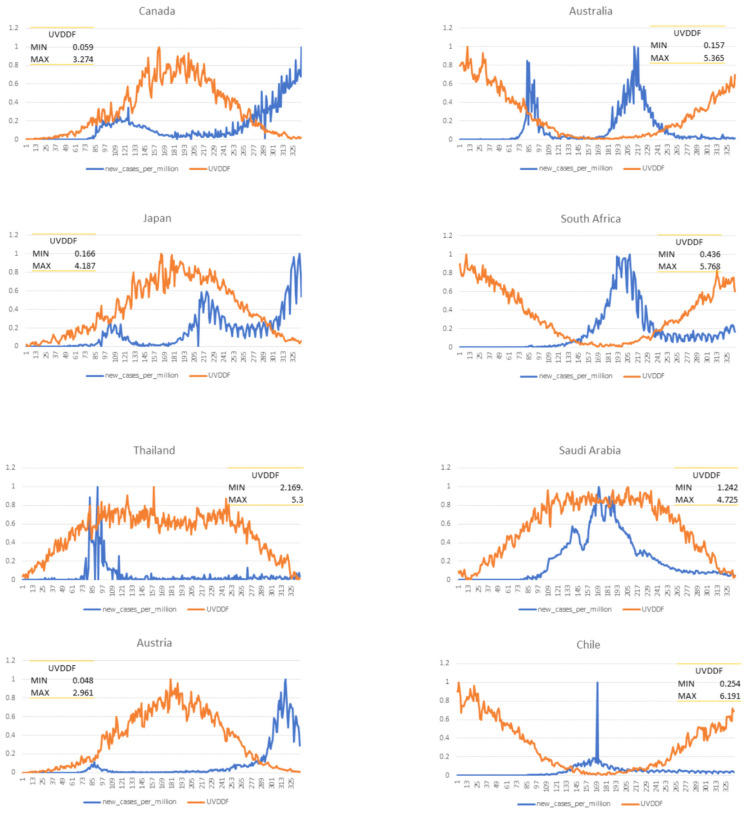

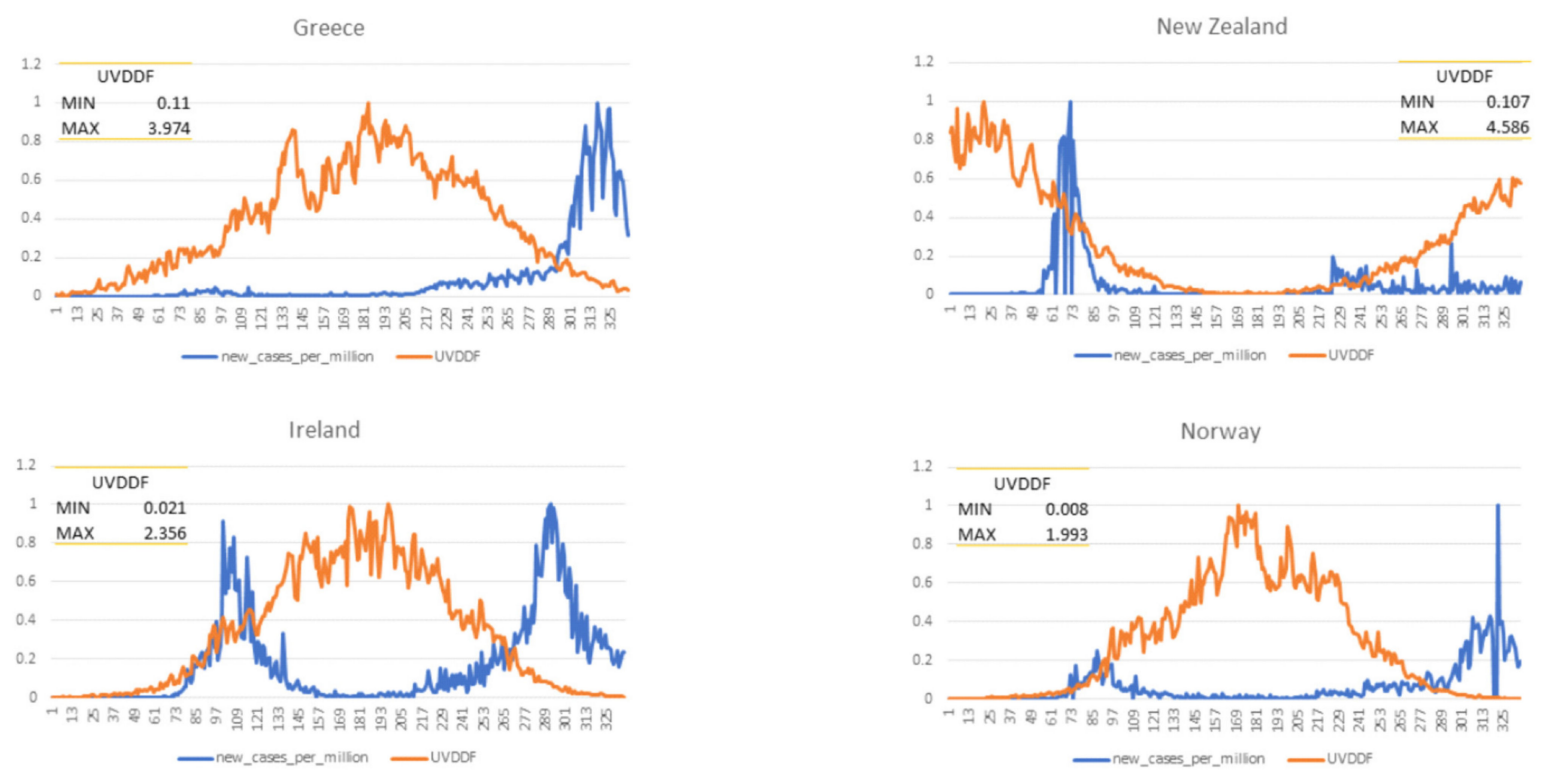
Time course of UVDDF and Covid-19 cases across target countries. Days are reported for the period 1 January–30 November (*x*-axis) and UVDDF levels (min-max normalized; *y*-axis, orange line). Covid-19 cases are reported (min-max normalized, *y*-axis, blue line). Absolute minimum and maximum values of UVDDF are also shown for each country, within panel.

Next, in order to demonstrate more clearly the seasonality parameter that UV radiation levels yield against daily Covid-19 cases, despite the restrictive measures imposed, we excluded mobility data as well as the UVDVF measure, focusing exclusively on the time-dependent quotidian-dependent course of the epidemic versus UVDDF levels (Figure 4). These simple two-dimensional graphs illustrate the evolution of ultraviolet radiation and new daily cases in each of the twelve target countries. The two model parameters that capture the essence of the epidemic by seasonality criteria emerged vividly. For several Northern Hemisphere countries, new cases peaked between days 70–120 against the background of mobility restrictions, followed by a significant drop over the local summer with a huge leap in increased mobility. For instance, in Greece, mobility was down to −80% in mid-March until early May, while it has increased to +140% in mid-July until September, against a baseline value in January 2020—then, cases surged from 8 to 10-fold in early October, compared to summer. The other nonpaired country, Ireland, had a surge of cases due to local situations that have been virtually eliminated, and then followed the same pattern. The paired countries, Canada, Japan, Austria and Norway, also followed these trends, contrasted to their Southern Hemisphere counterparts—Australia, South Africa, New Zealand and Chile—respectively (Figure 4). Finally, the control quasi-equatorial countries showed a dampened distribution, especially Thailand with Saudi Arabia “missing” the UV effect perhaps due to an indoors lifestyle during the harsh local summer. Thus, deleting the redundant UV (UVDVF) and mobility data, the association between UV as a seasonal index and Covid-19 cases as the conditional parameter emerged, strongly supporting seasonality for Covid-19.

It is evident that the observed patterns reflect a degree of seasonality seen here for the very first time over the year 2020. The achieved clarity is due to the availability of daily incidence data for the entire period of the Covid-19 epidemic and the normalization of the dependent model parameters (see Methods). The simple UV-daily case model implied an expected regular course of the pandemic that presented certain opportunities to optimize policy and use of NPIs until the situation is covered by extensive use of successful vaccines and pharmaceuticals. It can be argued that the most regular pattern was furnished by South Africa, as an “ideal”, hypothetical case for an epidemic curve that would be at a peak during the local winter of 2019, if such counts were hypothetically available (Figure 4).

**Figure 5:**
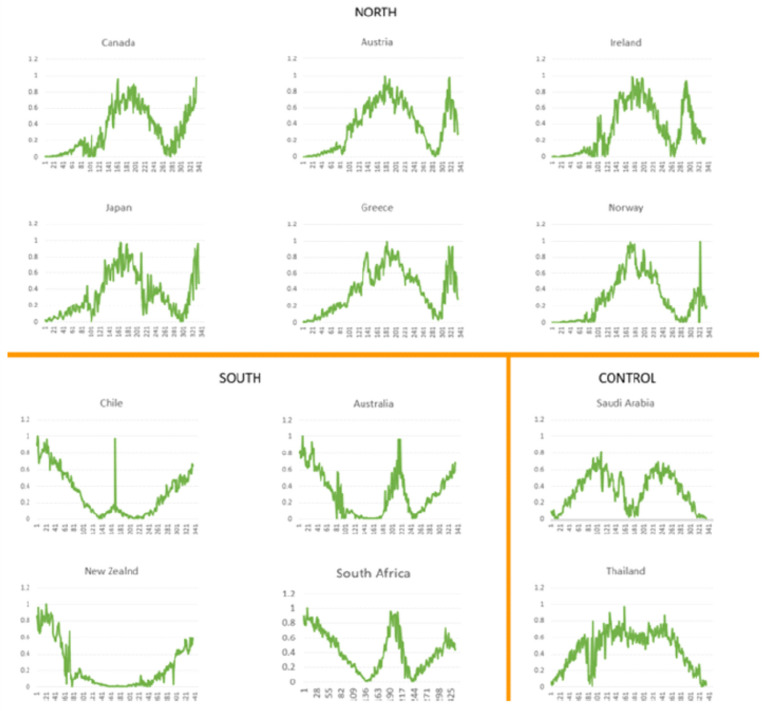
Absolute difference distributions between normalized daily cases and UV radiation levels. The twelve target countries are shown, the top half belonging to the Northern Hemisphere, the bottom right (two) being the (near-equatorial) control countries and the remaining bottom left (four) belonging to the Southern Hemisphere. The spike in the case of Chile is due to an irregularity of reporting backdated cases (see Methods). A smoothed average would have the side values more pronounced, resembling those of South Africa, despite different restrictive measures and other, unpredictable human factors or incidents. The SSQ metric (Table 2) can also be used as a summary statistic and a single-value comparative measure for all target countries.

The composite signal between the two model parameter curves over time could be obtained with absolute difference curves, as the values were min-max normalized and became fully comparable (see Methods), to depict seasonality patterns and their fluctuating behaviors for the target countries (Figure 5). The difference values unambiguously demonstrated the seasonality pattern of Covid-19 incidence rates in conjunction with UV radiation levels, as previously proposed [2]. A sum-of-squares metric over these single curves supplied a useful summary statistic, further amplifying the notion of similar trends across quite diverse countries, with a wide range of cultural, social, policy, geographical and epidemiological characteristics (Table 2). It remains to be seen whether the pattern will continue as we proceed into 2021, thus informing preventive and restrictive measures, in conjunction with other policies or progress in medication and vaccination. The patterns that are formulated by the more complex to the simpler yet equally robust indices lend support to the implicit model of UV-modulated Covid-19 seasonality. We sought to substantiate this claim by AI-based feature extraction and model interpretability via automated machine learning approaches.

The model received as input four model parameters, namely date, UVDDF level and mobility data as driving and walking statistics. The conditional parameter that needed to be predicted was Covid-19 daily case rate, using a multitude of potential machine learning models. Model optimization steps selected the best models that yielded the highest performance, i.e., minimal normalized square error. In our experience, the more effective models were the “voting ensemble” (with the exception of Australia, South Africa and Thailand) and the “stack ensemble” (for the three countries mentioned). We have found that these robust predictions with respect to the rather simple model structure and rich, complete input data were able to assert the impact of UV levels as a seasonality indicator that was both reliable and highly regular, compared to any other environmental parameter. In some sense, the application of machine learning to substantiate the hypothesis and interpretation of seasonality for Covid-19 represents a software-based reverse engineering strategy that confirmed both our hypothesis [2] and similar results obtained by different yet complementary approaches [7,15].

## 4. Discussion

Multiple factors are likely to influence Covid-19 incidence rates, first and foremost, human behavioral patterns as reflected either in individual mobility and social etiquette or in collective countermeasures such as business or school closures and other public health recommendations [27]. These measures, however, have been and keep being implemented without reference to a potentially key element of seasonality of the global epidemic. Conflicting reports on the possible influence of “weather” on Covid-19 spread dynamics have dispersed any slim chances we have had so far to follow seasonal cycles and use them to our advantage. The most critical characteristic of seasons is the day length period and not the weather: this partly explains the derailing of seasonality assessments with the wrong focus on temperature or humidity—and unproductive outcomes.

By selecting target countries which are, to a great extent, due to travel restrictions, self-contained “bubbles” where the epidemic unfolded, the geographical and socioeconomic affinities took out any demographic and cultural differences to a significant extent, as shown by the filter parameter step. Then, the seasonality effects of Covid-19 were exclusively dealt with using UV radiation levels and mobility information that reflected the period of the year and restrictive measures, respectively (Figure 1). It is thus surprising that, with such a simple formulation, the UV measurements and Covid-19 incidence exhibited starkly similar patterns for analogous countries throughout the year 2020 and in a quotidian-independent fashion (Figure 2). This effect was further demonstrated when mobility data were taken into account and showed that incidence rates were expectedly influenced with some lag and a few exceptions (Figure 3); and yet, UV radiation levels dominated the incidence patterns as an independent variable. Any behavioral patterns that are in fact affected by a winter-summer cycle can be accounted for in the seasonality element (*viz*. UV level) and not in the mobility factor, the latter being independent of any natural cycle for 2020. This was even more evident when only UV levels and incidence rates were compared for North-equatorial-South target countries (Figure 4). Despite the filtering step that resulted in certain similarities of geography and demography, these countries were sufficiently distinct—with different restrictive measures implemented in an asynchronous manner, cultural differences and age groups—so that the resulting modeling step presented a convincing case for a potential seasonality attribute for Covid-19 throughout 2020 and in the future. It is worth noting that various exceptions can be accounted for by unforeseen incidents (e.g., cruise ships in Australia) or local conditions (e.g., extensive indoor air-conditioning use in Saudi Arabia). The most well-behaved case in this framework might be South Africa, as it exhibited low incidence and high UV during the initial start of the pandemic, “as if” it was already conditioned and in recess after a winter outbreak (which, of course, was not there, around August 2019) (Figure 4).

**Figure 6:**
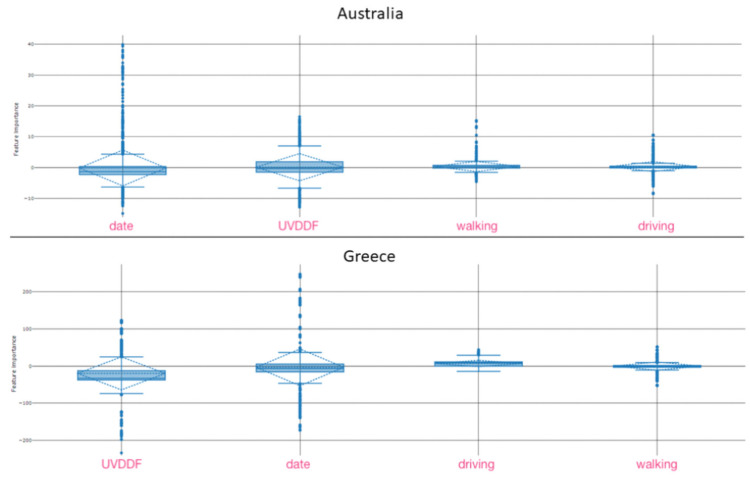
Examples of critical feature extraction for the model parameters in two target countries. Global feature importance was detected by meta-explainers (see Methods) that ranked the selected four model parameters against the conditional parameter, Covid-19 daily case rate. For Australia, date was followed by UVDDF and then mobility parameters; for Greece, UVDDF was followed by date and then mobility parameters. For details, see Methods. Full data for all target countries are available as Supplementary Material.

Our approach took advantage of open datasets about demographics, epidemiology, mobility and meteorology and benefits from a near real-time update of these dynamic datasets. The methodology is general enough that could be applicable to the assessment of seasonality for other respiratory infectious diseases. Both the quotidian-independent (Figure 2) and quotidian-dependent (Figure 4) pictorial representations have contributed to the execution of learning models that have extracted the feature importance of these time series automatically (Figure 6). The strong correlation of UV levels and Covid-19 incidence should not be taken as a causality factor, although this remains to be seen. From a biological perspective, seasonal conditions with varying day length and UV levels might modulate virus spread in outdoor situations, boost immune systems in hosts and influence other transmission dynamics. Yet, seasonality must be reflected according to day length variation and latitude, an effect demonstrated emphatically for Covid-19 throughout 2020 for ten nonequatorial countries.

The absolute difference distribution curves integrated UV level and Covid-19 data and unequivocally provided a strong argument for Covid-19 seasonal attributes, supported by machine learning, for very different countries and their restrictive measures, conditioned only on their location around the globe (Figure 5). As argued elsewhere, both the causative agent of Covid-19 (SARS-CoV-2) and human host behavior yield appropriate characteristics through their involvement in the present pandemic that it is reasonable to assume a seasonality for Covid-19 (Merow and Urban, 2020). Given the discovered patterns and modulation of Covid-19 incidence rates by UV radiation levels, NPIs should take seasonality into serious consideration to mitigate outbreaks in the coming months or years ahead. So far, restrictive measures have not worked too effectively, at least in Western countries where only partial lockdowns have been possible. The situation was exacerbated during the winter period of 2020–2021 in North America and Europe, indicating that the high level of transmissibility of the virus in winter conditions counteracts the little impact that imperfect restrictive measures might have. The timing of these measures was indeed reactive to epidemiological parameters only and not proactive in the absence of better understanding for the assessment of the seasonality element of the pandemic. We hope that in the near future, more insights will be gained for the management of Covid-19 by following the Earth’s seasonal cycles.

## Figures and Tables

**Table 1:** Countries and feature selection, example of top 10 entries. Snapshot of list of 51 countries processed for similar characteristics using filter parameter clustering. Columns: countries (name), coast/area (ratio), GDP (in US$), literacy (percentage), density (pop/km^2^), tests pM (coronavirus tests). Column names starting with “n” signify min-max normalization of the corresponding attribute. Available as Data Supplement 1 (DS01).

Id	Countries	Coast/Area	nCoast	GDP	nGDP	Literacy	nLit	Density	nDen	testspM	nTest
1	Albania	1.26	0.07	4500	0.08	86.5	0.77	100	0.15	2696	0.05
2	Australia	0.34	0.02	29,000	0.76	100	1.00	3	0.00	21,020	0.51
3	Austria	0	0.00	30,000	0.79	98	0.97	106	0.16	26,601	0.64
4	Belarus	0	0.00	6100	0.13	99.6	0.99	46	0.07	17,041	0.41
5	Belgium	0.22	0.01	29,100	0.76	98	0.97	376	0.57	19,000	0.46
6	Bolivia	0	0.00	2400	0.02	87.2	0.79	10	0.01	496	0.00
7	Brazil	0.09	0.01	7600	0.17	86.4	0.77	25	0.03	1597	0.03
8	Bulgaria	0.32	0.02	7600	0.17	98.6	0.98	63	0.09	3886	0.08
9	Canada	2.02	0.12	29,800	0.78	97	0.95	4	0.00	19,999	0.48
10	Chile	0.85	0.05	9900	0.23	96.2	0.94	23	0.03	8692	0.20

**Table 2:** The three groups of 12 countries in N/S Hemispheres. Group 1: large countries, Group 2: control (“c”, quasi-equatorial) countries, Group 3: smaller countries. The countries as exceptions (Saudi Arabia for indoors during summer, New Zealand for cruise ships and harsh restrictive measures are marked by an asterisk (*)—otherwise they would match Thailand and Austria, respectively). Added (non-equivalenced) countries are marked by a plus sign. All other countries in North (n) or South (s) are equivalenced, with the summary statistic sum of squares (SSQ) exhibiting similar values (irrespective of the shape of the normalized difference distribution). Available as Data Supplement 2 (DS02).

Group 1	SSQ	Region	Group 2	SSQ	Region	Group 3	SSQ	Region
Australia	53.82	n	Saudi Arabia *	56.09	c	Austria	75.03	n
Canada	58.98	s	Thailand	87.78	c	Chile	55.96	s
Japan	60.87	n				Greece +	77.96	n
South Africa	78.90	s				Ireland +	68.87	n
						New Zealand *	45.31	s
						Norway	54.48	n

## Data Availability

https://osf.io/kgxuf/, accessed on 18 February 2020, as Data Supplements DS01 (https://osf.io/nz29t/) and DS02 (https://osf.io/n6wdy/).
